# Editorial: MicroRNAs in Heart Regeneration: Regulatory Mechanisms and Therapeutic Applications

**DOI:** 10.3389/fcvm.2022.863332

**Published:** 2022-02-28

**Authors:** Eltyeb Abdelwahid, Katherine Athayde Teixeira de Carvalho

**Affiliations:** ^1^Feinberg Cardiovascular Research Institute, Feinberg School of Medicine, Northwestern University, Chicago, IL, United States; ^2^Advanced Therapy and Cellular Biotechnology in Regenerative Medicine Department, The Pelé Pequeno Príncipe Research Institute, Child and Adolescent Health Research and Pequeno Príncipe Faculties, Curitiba, Brazil

**Keywords:** miRNAs, regeneration, heart, therapy, cardiac repair

Heart diseases are major contributors to global health problems due to their high morbidity and mortality rates (1). MicroRNAs (miRNAs) are small, non-coding RNAs that have been considered potent post-transcriptional regulators of gene expression. The mechanisms regulating miRNAs are still incompletely understood; however, tremendous progress has been accomplished in revealing fundamental pathways and attractive therapeutic targets for enhancing heart regeneration and repair, over the past few years. Disrupted regulation of miRNAs expression occurs in many cardiovascular pathologies, leading to cardiac remodeling and progression to heart failure. Delineation of molecular pathways controlling miRNAs and their effects on cardiac tissue requires an understanding of essential mechanisms of cardiomyocytes regeneration (2–4). Recent discoveries have identified many miRNAs with beneficial roles in essential aspects including cardiac development, proliferation, migration, stem cell self-renewal, differentiation and function of stem cells, and heart regeneration, thus allowing improvement of the current strategies for cardiac therapies (4). Emerging progress in material science and nanotechnology has improved the research of methods for delivering miRNAs. Overall, the therapeutic application of miRNAs has been shown to be protective in experimental models of cardiac disease and in some clinical trials. This Research Topic focuses on the role of miRNAs in various cardiac problems such as congenital heart abnormalities, aging and adult heart diseases, with special emphasis on regulatory mechanisms and potential therapeutic applications. The articles in this Research Topic reflect the efforts made to provide high-quality data that could broaden the knowledge of the role of miRNAs in heart pathologies.

Transposition of the great arteries (TGA) is a most severe congenital heart disease (CHD), but understanding its etiology, morphogenesis, molecular, biomarkers, and embryological mechanisms await comprehensive research (5). Abu-Halima et al. showed that in patients with transposition of the great arteries and a systemic right ventricle (TGA-RV), miR-183-3p is an independent predictor of worsening cardiac failure and thus may be used as an additional biomarker in the risk evaluation of these patients. These results indicate potential therapeutic links between TGA and miRNAs implicated in complex congenital heart disease associated with heart failure. Further progress in application of miRNAs in the treatment of cardiac anomalies require in-depth pre-clinical studies on common genetic mechanisms regulating normal and abnormal anatomical aspects both in animal models and humans. Yang et al. studied the differential expression of miR-153-3p in Formaldehyde (FA) treated embryonic cardiomyocytes and in an animal model of fetal cardiomyocyte apoptosis. The results indicated that βII spectrin regulates FA-induced cardiomyocyte apoptosis. The authors showed that miR-153-3p targets βII spectrin to negatively affect myocardial development. The levels of apoptosis markers were consistent both *in-vitro* and *in-vivo*. Interestingly, FA triggers myocardial fibrosis during heart development. This paper provides new insights into the regulatory mechanisms regulating cardiomyocyte apoptosis in CHD. It is thus suggested that miR-153-3p may be a potential target for CHD therapies. Rusu-Nastase et al. conducted experiments to identify miRNAs with potential therapeutic value for age-associated cardiac fibrosis. The results indicated that miR-29a level increases in multiple organs in the natural aging process and it may represent a compensatory mechanism against heart fibrosis *via* SERPINH1 downregulation. Further investigations may explain detailed molecular aspects contributing to the protective role of miR-29a in the aging process. miRNAs are regarded as a major part of the development of cardiac dysfunction. The diverse data on differential miRNA regulation may allow identifying their impact on cardiovascular disease both by changing the repair potential and by modulating cellular function and phenotype.

Zhou et al. reviewed various types of chemical modifications and viral-vector-based delivery systems used for miRNA modulation in HF. The authors discussed the opportunities and challenges for miRNAs as targets for the treatment of HF. Besides, they discussed CDR132L as a first miRNA drug utilized to treat HF. Accumulating knowledge on the effect of inappropriate expression of miRNAs has improved the understanding of different heart pathologies as well as uncovered miRNAs that can be potential targets for enhancing effective stem cell therapy. These studies should improve repair strategies such as stem cell transplantation, gene therapy, and reprogramming. Effective basic and clinical research requires solving obstacles resulting from an incomplete understanding of disease-related complex regulatory networks as well as inadequate availability of technologies. Importantly, comprehensive research on development of novel delivery technologies is needed for successful translational research in preclinical and clinical settings. Nevertheless, the concept of administering a combination of miRNAs to target different pathways awaits further assessment in cardiac cells. The discovery of miRNA inhibitors/activators such as miR-mimics, antagomiRs, and decoys to regulate miRNAs function has proposed using them as potential therapeutic tools for the treatment of heart failure conditions. However, various challenges need to be solved before applying them clinically such as specificity, half-life, efficiency, and route of administration.

In summary, this Research Topic addressed emerging discoveries on the potential roles of MiRNAs in heart diseases at the basic scientific and clinical levels. The current progress in the field should pave the path for new therapeutic strategies and tools for designing appropriate MiRNA-based treatments for regenerating and repairing the heart.

## Author Contributions

Both authors listed have made a substantial, direct, and intellectual contribution to the work and approved it for publication.

## Conflict of Interest

The authors declare that the research was conducted in the absence of any commercial or financial relationships that could be construed as a potential conflict of interest.

## Publisher's Note

All claims expressed in this article are solely those of the authors and do not necessarily represent those of their affiliated organizations, or those of the publisher, the editors and the reviewers. Any product that may be evaluated in this article, or claim that may be made by its manufacturer, is not guaranteed or endorsed by the publisher.
